# The Complex Interplay Between Physical Activity and Recovery Styles in Patients With Severe Mental Disorders in a Real-World Multicentric Study

**DOI:** 10.3389/fpsyt.2022.945650

**Published:** 2022-07-11

**Authors:** Gaia Sampogna, Mario Luciano, Matteo Di Vincenzo, Ileana Andriola, Enrico D'Ambrosio, Mario Amore, Gianluca Serafini, Alessandro Rossi, Claudia Carmassi, Liliana Dell'Osso, Giorgio Di Lorenzo, Alberto Siracusano, Rodolfo Rossi, Andrea Fiorillo, Working Group LIFESTYLE, Vincenzo Giallonardo

**Affiliations:** ^1^Department of Psychiatry, University of Campania “L. Vanvitelli”, Naples, Italy; ^2^Department of Basic Medical Science, Neuroscience, and Sense Organs, University of Bari Aldo Moro, Bari, Italy; ^3^Department of Neuroscience, Rehabilitation, Ophthalmology, Genetics, Maternal and Child Health (DINOGMI), Section of Psychiatry, University of Genoa, Genoa, Italy; ^4^Department of Biotechnological and Applied Clinical Sciences (DISCAB), University of L'Aquila, L'Aquila, Italy; ^5^Psychiatric Unit, Department of Clinical and Experimental Medicine, AOUP, University of Pisa, Pisa, Italy; ^6^Department of Systems Medicine, University of Rome Tor Vergata, Rome, Italy

**Keywords:** lifestyle, physical activity, sedentary behaviors, mortality, severe mental disorders

## Abstract

Compared with the general population, people with severe mental disorders have significantly worse physical health and a higher mortality rate, which is partially due to the adoption of unhealthy lifestyle behaviors, such as heavy smoking, use of alcohol or illicit drugs, unbalanced diet, and physical inactivity. These unhealthy behaviors may also play a significant role in the personal and functional recovery of patients with severe mental disorders, although this relationship has been rarely investigated in methodologically robust studies. In this paper, we aim to: a) describe the levels of physical activity and recovery style in a sample of patients with severe mental disorders; b) identify the clinical, social, and illness-related factors that predict the likelihood of patients performing physical activity. The global sample consists of 401 patients, with a main psychiatric diagnosis of bipolar disorder (43.4%, *N* = 174), psychosis spectrum disorder (29.7%; *N* = 119), or major depression (26.9%; *N* = 118). 29.4% (*N* = 119) of patients reported performing physical activity regularly, most frequently walking (52.1%, *N* = 62), going to the gym (21.8%, *N* = 26), and running (10.9%, *N* = 13). Only 15 patients (3.7%) performed at least 75 min of vigorous physical activity per week. 46.8% of patients adopted sealing over as a recovery style and 37.9% used a mixed style toward integration. Recovery style is influenced by gender (*p* < 0.05) and age (*p* < 0.05). The probability to practice regular physical activity is higher in patients with metabolic syndrome (Odds Ratio - OR: 2.1; Confidence Interval - CI 95%: 1.2–3.5; *p* < 0.050), and significantly lower in those with higher levels of anxiety/depressive symptoms (OR: 0.877; CI 95%: 0.771–0.998; *p* < 0.01). Globally, patients with severe mental disorders report low levels of physical activities, which are associated with poor recovery styles. Psychoeducational interventions aimed at increasing patients' motivation to adopt healthy lifestyle behaviors and modifying recovery styles may improve the physical health of people with severe mental disorders thus reducing the mortality rates.

## Background

Recovery is a “process of change through which individuals improve their health and wellness, live a self-directed life, and strive to reach their full potential”. In patients with severe mental disorders, recovery should represent the final goal of personalized treatment plans for all clinicians (1–3). The recovery styles adopted by patients with severe mental disorders predict their personal and psychosocial functioning as well as their adherence to therapeutic plans. McGlashan et al. (4) described a continuum of recovery styles, from “sealing over”, which is characterized by avoiding the illness experience and is associated with negative long-term outcomes (5), to “integration”, characterized by incorporating the psychotic episode into own identity. The integration style is associated with better long-term outcomes, in terms of adherence to treatments and engagement in psychosocial interventions (6–8).

The full recovery of people with severe mental disorders is hampered by many clinical and socio-demographic factors, including patient's age, pre-morbid level of functioning, educational level, working condition, social network, cognitive schemas (9), severity and type of symptoms, duration of illness, level of insight (10), clinical staging, previous treatments, time to remission, patient's social network, family ties, environmental exposures, presence of physical comorbidities (11, 12). In particular, patients with severe mental disorders have very poor physical health, suffering from coronary heart diseases, diabetes, respiratory, renal, and infectious diseases (13–17). The higher presence of physical illnesses compared to the general population is due to several causes (18, 19), including the adoption of unhealthy lifestyle behaviors, such as heavy smoking (20–23), heavy alcohol drinking (24–26), use of illicit drugs (27, 28), unbalanced diet and low levels of physical activity (29).

Physical activity, which is defined as “any bodily movement produced by skeletal muscles that results in energy expenditure”, and physical exercise, defined as “a subset of physical activity that is planned, structured, and repetitive and has as a final or an intermediate objective the improvement or maintenance of physical fitness” (30), have positive effects on both physical and mental health (31). In fact, people who perform regular physical activity have a reduced risk of all-cause mortality and cardiovascular mortality.

Interventions increasing the levels of physical activity improve body composition and cardiorespiratory fitness and reduce the cardiometabolic burden and psychiatric symptoms (32). They also improve patients' quality of life, cognition, personal functioning, life skills, and social networks (33–35). Moreover, by improving patients' self-confidence and motivation to change, physical activity is also associated with improvement in the recovery process.

The physical health of patients with severe mental disorders is too often devaluated (36–39). Clinicians worldwide tend to prioritize other (mental) health domains, with the consequence of not motivating enough their patients toward physical activity (40–42). The clinical, biological, and social correlates of physical activity in people with severe mental disorders have been investigated only in a few studies (43, 44). Moreover, studies exploring the relationship between physical activity and recovery styles are also lacking (45).

Appropriate interventions increasing the levels of physical activity of patients with severe mental disorders should be developed (46, 47). Several trials have been promoted with a specific focus on physical activity, including a motivational coaching in order to increase the participation in physical activity programmes of patients with severe mental disorders (48–50).

In this paper, we: (a) describe the levels of physical activity and the recovery styles in patients with severe mental disorders; (b) investigate the clinical, social, and illness-related factors that are associated with the levels of physical activity in patients with severe mental disorders.

## Methods

The present paper is based on data collected within the LIFESTYLE trial (51), a national, multicentric, randomized, controlled trial with blinded outcome assessments, coordinated by the University of Campania “Luigi Vanvitelli” in Naples and carried out in collaboration with Universities of Bari, Genova, L'Aquila, Pisa, and Rome-Tor Vergata.

Patients were included in the study if they met the following criteria: (1) age between 18 and 65 years; (2) diagnosis of schizophrenia, schizoaffective disorder, delusional disorder, other psychotic disorder, major depressive disorder, or bipolar disorder according to the DSM-5 and confirmed by the Structured Clinical Interview for DSM-5 (SCID-5); (3) ability to provide written informed consent; (4) BMI ≥ 25; (5) in charge to the local mental health center at least for three months before recruitment.

The main outcome measure considered for this analysis is the level of physical activities. Physical activity has been evaluated with the International Physical Activity Questionnaire (IPAQ)—short form (52), which is a 18-item questionnaire exploring physical activity in terms of walking, moderate-intensity, and vigorous-intensity physical activities.

The 24-items Questionnaire on lifestyle behaviors, developed by the Italian National Institute of Health, has been used to explore patients' dietary patterns (e.g., food eaten at lunch or dinner), smoking habits (e.g., number of cigarettes smoked per day; attempts to quit smoking), and physical activity (e.g., time spent in walking per day) (53).

Recovery styles have been evaluated with the Recovery Style Questionnaire (RSQ) (54), a 39-item self-report assessment instrument exploring six styles of adaptation to severe mental disorder and recovery: “sealing over”, “tends toward sealing over”, “mixed picture in which sealing over predominates”, “mixed picture in which integration predominates”, “tends toward integration”, and “integration”.

Other assessment tools include the Food Frequency Questionnaire - short version (55); the Fagerström Test for Nicotine Dependence (FTND) (56); the Pittsburgh Sleep Quality Index (PSQI) (57); the Leeds Dependence Questionnaire (LDQ) (58); the Morisky Medication Adherence Scale (MMAS) (59); the Cumulative Illness Rating Scale (CIRS) (60); the Manchester Short Assessment of Quality of Life (61); the Measurement and Treatment Research to Improve Cognition in Schizophrenia (MATRICS) Consensus Cognitive Battery (MCCB)—brief version (62, 63); the Internalized Stigma of Mental Illness (ISMI) (64); *ad-hoc* questionnaire on sexual health; the Pattern of Care Schedule (PCS)—modified version (51).

Information on weight, height, BMI, waist circumference, blood pressure, resting heart rate, HDL, LDL and overall levels of cholesterol, blood glucose, triglycerides, and blood insulin have been collected by the researcher with the Anthropometric schedule. The homeostatic model assessment (HOMA) index and the Framingham Risk Score have been calculated for quantifying insulin resistance and cardiovascular risk, respectively.

Patients' psychopathological status has been assessed with the 24-item Brief Psychiatric Rating Scale (BPRS) (65). Patients' social functioning has been explored through the Personal and Social Performance Scale (66), a 100-point single-item rating scale, subdivided into four main areas: (1) “socially useful activities”; (2) “personal and social relationships”; (3) “self-care”; and (4) “disturbing and aggressive behaviors”.

This study was conducted in accordance with globally accepted standards of good practice, in agreement with the Declaration of Helsinki and with local regulations. The study protocol was formally approved by the Ethics Committee of the Coordinating Center in January 2017 (Approval Number: 64). All other methodological details of the LIFESTYLE study are reported in (51) and the trial registration number is the following: 2015C7374S.

### Statistical Analyses

Descriptive statistics and frequency tables have been used to assess patients' socio-demographic and clinical characteristics. Chi-square with multiple comparisons and ANOVA with Bonferroni corrections have been adopted to detect differences in the levels of physical activities. Bivariate analyses have been performed in order to evaluate the association between the levels of physical activities, the recovery styles, and the severity of clinical symptoms.

Multivariate logistic regression models have been implemented to identify predictors of practicing physical activity. The models have been adjusted for several socio-demographic characteristics, including gender, age, presence of physical illness, being married, level of education, satisfaction with one's own life, adaptive and maladaptive coping strategies, duration of illness, and recovery styles. This statistical approach has been already adopted in previous published papers based on the LIFESTYLE trial (51, 67, 68) and the categorical variable “Center” was also entered in the regression model.

A multiple imputation approach has been used for managing missing data. The level of statistical significance was set at *p* < 0.05 and statistical analyses were performed using the Statistical Package for Social Sciences (SPSS), version 26.0, and STATA, version 15.

## Results

### Patients' Socio-Demographic and Clinical Characteristics

The final sample consists of 401 patients, mostly female (57.1%, *N* = 229) and with a mean age of 45.6 (±11.8) years. Patients are affected by bipolar disorder (43.4%, *N* = 174), psychosis spectrum disorder (29.7%; *N* = 119), or major depression (26.9%; *N* = 118); the duration of the illness is about 15.6 (±11.3) years.

Most patients present mild symptoms at the BPRS and have a discrete level of personal functioning (PSP value: 65.7 ± 15.1).

Most patients are either overweight (35.4%; *N* = 142; BMI ranging between 25–29.9) or obese (34.9%; *N* = 140), with a mean BMI of 32.2 (±5.5). 40.6% of patients are heavy smokers and 67.8% drink alcohol more than three times per week. 53.4% of patients (*N* = 214) suffer from the metabolic syndrome; in particular, 56.6% (*N* = 227) have systolic hypertension, 36.1% (*N* = 143) diastolic hypertension and 26.9% (*N* = 108) hyperglycaemia.

As regards diet habits, 31.0% of patients eat less than two portions of fruits per week and 37.6% less than two portions of vegetables per week. 43.5% of the sample drink about one liter of water per day, below the WHO recommended threshold of more than two liters/day (Table 1).

**Table 1:** Socio-demographic and clinical characteristics of the sample.

	**Global sample (*****N*** **=** **401)**		**Practicing regular physical activity**				
			**Yes (*N =* 118)**		**No (*N =* 283)**		***P-*value**
**Socio-demographic variables**							
Gender, Female, % (*N*)	57.1 (229)		51.7 (51)		59.4 (168)		NS
Age group, % (*N*)							0.001
18–34 years	19.2 (77)		27.1 (32)		15.9 (45)		
Age 35–64	79.1 (317)		67.8 (80)		83.7 (237)		
Over 65	1.7 (7)		5.1 (6)		0.4 (1)		
Occupational status, employed, % (*n*)	46.1 (185)		44.9 (53)		46.6 (132)		NS
DSM-5 diagnosis, % (*N*)							NS
Psychosis spectrum disorder	29,7 (119)		37.3 (44)		26.5 (75)		
Bipolar disorder	43.4 (174)		39.0 (46)		45.2 (128)		
Depressive disorder	26.9 (108)		23.7 (28)		28.3 (80)		
Duration of the illness, years M (SD)	15.6 (11.3)		15.0 (11.2)		15.9 (11.4)		NS
	**Mean**	**SD**	**Mean**	**SD**	**Mean**	**SD**	
**Clinical variables**							
**Brief psychiatric rating scale**							
Global score	5.4	2.0	5.3	1.9	5.4	2.1	NS
Anxiety/depressive symptoms	8.8	3.1	8.0	3.2	9.1	3.1	0.003
Anergia	7.7	3.17	7.0	2.8	7.9	3.2	0.004
Hyperactivity symptoms	4.7	1.9	4.8	1.8	4.7	1.9	NS
Hostility symptoms	4.0	1.9	3.7	1.5	4.2	1.9	0.036
Personal functioning, global score	65.7	15.1	66.1	14.6	65.4	15.4	NS
Adherence to treatment	1.1	1.0	0.9	0.9	1.1	1.1	NS
Levels of internalized stigma	10.9	2.1	10.7	2.2	11.1	2.1	NS
Quality of life, global score	4.1	1.0	4.3	0.9	3.9	1.1	NS
MATRICS—Bacs scoring	36.9	50.3	34.7	13.9	37.9	58.9	NS
MATRICS—Category fluency	17.9	5.4	17.6	5.3	18.0	5.5	NS
MATRICS—Trial Making Test	52.4	28.6	52.7	24.0	52.3	29.9	NS
	***%***	***N***	***%***	***N***	***%***	***N***	
Typical antipsychotic	21.2%	(85)	21.2%	(25)	21.2%	(60)	NS
Atypical antipsychotic	59.6%	(239)	62.7%	(74)	58.3%	(165)	NS
Mood stabilizer	54.9%	(220)	56.2%	(159)	51.7%	(61)	NS
Benzodiazepines	46.6%	(187)	47.0%	(133)	45.8%	(54)	NS
Tricyclic antidepressant	5.7%	(23)	6.8%	(8)	5.3%	(15)	NS
Second generation antidepressant	46.4%	(186)	42.4%	(50)	48.1%	(136)	NS
**Lifestyle behaviors**	**%**	***N***	**%**	***N***	**%**	***N***	
Smoker, yes	40.6%	(163)	37.3%	(44)	42%	(119)	NS
Fruit intake, three or more times/week	69%	(249)	73.4 %	(80)	67.1%	(169)	NS
Vegetable intake, three or more times/week	62.4%	(227)	59.3%	(64)	63.7%	(163)	NS
Water consumption daily, at least two liters	39.5%	(158)	39%	(46)	39.7%	(112)	NS
**Metabolic parameters**	**Mean**	**SD**	**Mean**	**SD**	**Mean**	**SD**	
Systolic blood pressure, mmHg	125.6	13.5	124.1	12.8	126.3	13.8	NS
Diastolic blood pressure, mmHg	80.7	8.9	79.8	7.5	81.1	9.5	NS
Waist circumference, cm	109.3	14.0	106.6	13.6	110.4	14.1	0.015
Glycemia, mg/dl	95.4	27.0	94.2	22.9	95.9	28.6	NS
Insulinemia, microU/ml	17.4	18.3	15.1	11.5	18.3	20.5	NS
Triglycerides, mg/dL	171.2	129.7	177.7	152.6	168.5	119.0	NS
Total cholesterol, mg/dL	189.9	40.9	189.4	38.9	190.0	41.8	NS
Low density lipoproteine, mg/dL	119.2	34.9	118.2	32.4	119.6	36.0	NS
High density lipoproteine, mg/dL	46.0	14.6	44.9	12.1	46.5	15.6	NS
HOMA index	4.9	11.6	3.8	3.7	5.4	13.6	NS
Framingham risk score	9.8	4.5	9.4	5.1	9.9	4.2	NS
Framingham risk score- −10 Years	9.3	7.5	9.3	7.9	9.4	7.4	NS
Metabolic Syndrome	53.4%	(214)	45.8%	(54)	56.5%	(160)	0.049
BMI, M (SD)	32.5	5.5	31.3	4.9	33.0	5.7	0.004

*NS, Not significant*.

### Recovery Styles and Levels of Physical Activity

**Figure 1:**
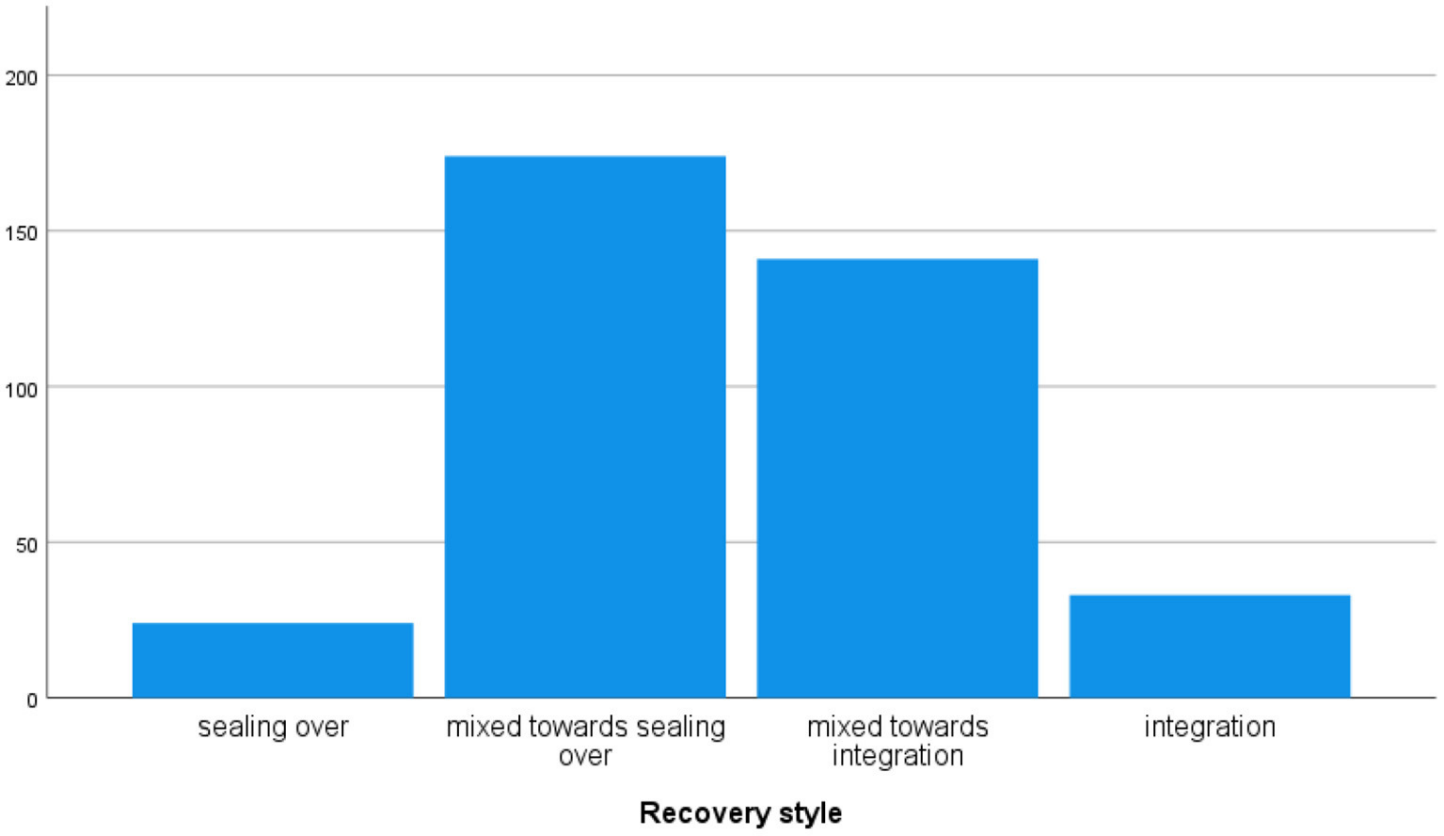
Recovery style in the global sample.

The “sealing over” recovery style is adopted by 46.8% of patients (*N* = 174), a “mixed style” is used by 37.9% of patients (*N* = 141), and “integration” is used by 8.9% of patients (*N* = 33) only (Figure 1). Recovery styles vary according to gender (*p* < 0.05) and age (*p* < 0.05), while there are no differences according to the diagnostic category. Patients adopting “integration” have lower levels of anxiety/depressive and hostility symptoms compared to those using “sealing over” (*p* < 0.030 and *p* < 0.050, respectively) (Table 2).

**Table 2:** Differences in recovery styles according to practicing regular physical activity and to symptoms' severity.

	**Practicing regular physical activity**				***P-*value**
	**Yes (*N = * 118)**		**No (*N = * 283)**		
	**%**	***N***	**%**	***N***	
Sealing over	5.8	6	6.7	18	NS
Mixed toward sealing over	48.1	50	46.3	124	
Mixed toward integration	35.6	37	38.8	104	
Integration	10.6	11	8.2	22	
Missing		14		15	
		**Mean**	**SD**	***P-*** **value**
BPRS Anxiety/Depressive symptoms	Sealing over^a^	9.8	2.7	0.030
	Mixed toward sealing over	9.1	3.2	
	Mixed toward integration	8.8	2.9	
	Integration^a^	7.6	2.8	
BPRS Anergia symptoms	Sealing over	8.2	3.3	0.365
	Mixed toward sealing over	7.8	3.2	
	Mixed toward integration	7.7	3.0	
	Integration	6.9	2.2	
BPRS Total symptoms	Sealing over	6.0	2.5	0.436
	Mixed toward sealing over	5.4	2.2	
	Mixed toward integration	5.3	1.8	
	Integration	5.5	2.2	
BPRS Hyperactivity symptoms	Sealing over	5.2	1.7	0.486
	Mixed toward sealing over	4.8	1.9	
	Mixed toward integration	4.7	1.7	
	Integration	4.5	1.7	
BPRS Hostility symptoms	Sealing over^b, c, d^	5.3	2.9	0.016
	Mixed toward sealing over ^d^	4.0	1.9	
	Mixed toward integration^b^	4.0	1.8	
	Integration^c^	3.9	1.3	

*Pairwise comparisons with Bonferroni corrections: ^a^p < 0.042; ^b^p < 0.014; ^c^p < 0.015; ^d^p < 0.040; NS, not significant*.

Regular physical activity is performed by 29.4% (*N* = 119) of patients. The most frequent physical activities performed by patients are walking (52.1%, *N* = 62), going to the gym (21.8%, *N* = 26), running (10.9%, *N* = 13), playing football (7.6%, *N* = 9), cycling (9.2%, *N* = 11), and swimming (2.5%, *N* = 3). Physical activities' preferences are not influenced by body mass index, age, and duration of illness. Only playing football is preferred by male patients (*p* < 0.001).

Vigorous physical activity performed for at least 75 min per week is done by 15 patients (3.7%), while moderate physical activity by 21.7% of patients (Table 3).

**Table 3:** Levels of physical activity evaluated at the IPAQ.

	**Global sample (*N* = 401)**	**Practicing regular physical activity**		
		**Yes (*N =* 118)**	**No (*N =* 283)**	***P-*value**
**Number of days practicing vigorous physical activity**				0.001
None	87.3% (350)	72.9% (86)	83.9%(99)	
At least 1 day	12.7 % (51)	27.1%(32)	16.1%(19)	
Performed at least 75 min of vigorous physical activity per week, yes	3.7% (15)	7.6% (9)	2.1% (6)	0.017
**Number of days practicing moderate physical activity**				0.001
None	78.3% (314)	52.5% (62)	89.0% (252)	
At least 1 day	21.7% (87)	47.5% (56)	11% (31)	
Number of days walking at least 10 min/days				0.002
Seven/seven	35.4 % (142)	45.8% (54)	31.1% (88)	

Patients performing regular physical activities have lower levels of anergia (7.0 ± 3.2 vs. 8.1 ± 2.8, *p* < 0.001) and hostility (4.2 ± 1.9 vs. 3.7 ± 1.5, *p* < 0.001) at the BPRS compared with those not practicing physical activities; no other significant clinical differences exist in the other clinical domains between the two groups. Patients practicing physical activity report higher levels of perceived satisfaction with the quality of life compared with non-active patients (4.3 ± 0.9 vs. 3.9 ± 1.1, *p* < 0.005). There are no differences in the levels of personal functioning, internalized stigma, treatment adherence, and cognitive functioning. The levels of physical activity do not differ according to the condition of being smokers or being alcohol drinkers (Table 1). No differences were found between those patients practicing regular physical activities and those not practicing it in the recovery styles.

### Multivariate Analyses

According to the multivariate logistic regression models, patients with metabolic syndrome have a higher probability to practice regular physical activity (OR: 2.1; CI 95%: 1.2–3.5; *p* < 0.050). Patients with higher levels of anxiety/depressive symptoms (OR: 0.877; CI 95%: 0.771–0.998; *p* < 0.01) have a significantly lower tendency to practice physical activity. The likelihood of practicing regular physical activity is not influenced by other lifestyle variables, including diet, smoking, or drinking water, as well as other illness-related variables, such as duration of illness, pharmacological treatment, and recovery style (Table 4).

**Table 4:** Predictors of practicing regular physical activity.

	***B***	**S.E**.	**Sign**.	**OR**	**95% CI**	
					**Lower bound**	**Upper bound**
**Lifestyle-related factors**						
Smoker	−0.302	0.293	0.302	0.739	0.417	1.312
Fruit intake	−0.181	0.463	0.697	0.835	0.337	2.068
Vegetables intake	−0.229	0.371	0.537	0.795	0.384	1.646
Water consumption daily	0.016	0.195	0.934	1.016	0.693	1.491
Metabolic Syndrome	0.718	0.279	**0.010**	**2.050**	**1.188**	**3.540**
**Illness-related factors**						
MANSA total score	−0.031	0.159	0.844	0.969	0.710	1.323
PSP total	0.002	0.011	0.891	1.002	0.980	1.024
BPRS hyperactivity	0.156	0.094	0.097	1.169	0.972	1.406
BPRS anxiety/depressive	−0.131	0.066	**0.046**	**0.877**	**0.771**	**0.998**
BPRS anergia	−0.078	0.066	0.241	0.925	0.812	1.054
BPRS hostility	−0.103	0.116	0.371	0.902	0.719	1.131
BPRS total	0.060	0.102	0.559	1.061	0.869	1.296
Atypical antipsychotic	−0.290	0.315	0.358	0.748	0.404	1.388
Typical antipsychotic	−0.330	0.361	0.361	0.719	0.355	1.458
Benzodiazepine	−0.232	0.288	0.420	0.793	0.451	1.393
Trycyclic antidepressant	−0.147	0.645	0.820	0.863	0.244	3.057
II gen. antidepressant	−0.220	0.317	0.487	0.802	0.431	1.492
Constant	3.630	2.305	0.115	37.717		

*Regression model has been controlled for possible confounding factors including age, gender, duration of the illness, and center*.

*Significant values have been highlighted using bold characters*.

## Discussion

Recovery from severe mental illness is a complex and multifaceted process, which represents the ultimate goal of a treatment plan for patients affected by different mental disorders. The adoption of different recovery styles by patients influences their personal and psychosocial functioning, therapeutic adherence, and treatments' engagement.

In our sample, the majority of recruited patients use a “sealing over” recovery style, which is associated with a negative long-term outcome (4). In fact, “sealing over” patients have an insecure identity (7), report negative experiences in early attachment, have social difficulties, and are affected by predominant negative symptoms. On the contrary, people adopting an “integration” style (i.e., incorporating the psychotic episode into their identity), report more favorable long-term outcomes in terms of engagement with services and perceived quality of life (69–71). A recent study carried out in Italy found that the integration style is associated with a good functional outcome, through acceptance of the psychotic experience and the awareness of the need for support and care, while patients adopting sealing over were less likely to maintain their social role and to invest in interpersonal relationships, with a global poorer long-term outcome (6). Unfortunately, in our study, only a minority of patients adopt this recovery style.

The present study is focused on recovery styles and the association with the levels of physical activities. In fact, recovery styles influence treatment engagement and illness status (45), as also the propensity to perform physical activities. On the other hand, physical activity can promote recovery by improving patients' self-confidence, health status, and motivation to change. In our sample, patients reported low levels of physical activities and low levels of recovery (characterized by a prevalence of sealing over style), confirming the bidirectional relationship between recovery and physical activity. It would be interesting to explore the effects of a physical activity intervention on the levels of recovery styles of patients with severe mental disorders in a longitudinal study.

A significant obstacle to recovery in people with severe mental disorders is represented by the high rate of physical comorbidities and the reduced life expectancy compared with the general population (72–76). Several factors contribute to the higher mortality and morbidity in patient with severe mental disorders, such as the higher prevalence rate of metabolic syndrome compared to the general population (77, 78) and the adoption of unhealthy lifestyle behaviors (79). In fact, patients with severe mental disorders are frequently physically inactive, not fulfilling the WHO recommendations (80, 81). Our findings confirm the low levels of physical activity in patients with severe mental disorders (29), with only one patient out of three reporting to perform any type of physical activity. Moreover, when considering the type of physical activity, only 3.7% of patients performed at least 75 min of vigorous physical activity per week, which is the WHO recommended threshold for having a beneficial impact on physical health. It may be that people do not even know what is considered “regular physical activity” (82), suggesting the need to develop and carry out informative interventions tailored to the general population and people with severe mental disorders. Within the LIFESTYLE project, our research team has developed a psychoeducational lifestyle group intervention for people with severe mental disorders (51), whose efficacy in the improvement of healthy lifestyle behaviors has been documented in a randomized controlled trial (67, 68).

The various socio-demographic variables considered did not influence the choice of any specific physical activity, differently from data collected in the general population (83). This finding confirms that it is not possible to simply translate the strategies developed for the general population to increase physical activities to people with severe mental disorders, but that more specific and targeted interventions are needed (84, 85).

In our regression models, lifestyle- and illness-related factors have been tested as possible predictors of practicing regular physical activity. The presence of the metabolic syndrome was the only lifestyle factor significantly predicting the likelihood of patients practicing physical activity, even after controlling for age, gender, duration of illness, and pharmacological treatments. Other lifestyle factors, such as diet or smoking, do not have any impact on the outcome. It may be that patients with severe mental disorders are reluctant to practice physical activities regularly and tend to do so only as a “last resort” when they are diagnosed with severe physical disturbances, such as hypertension or obesity, which are core elements of the metabolic syndrome. This finding suggests the need the improve regular physical check-up visits for patients with severe mental disorders, who are instead treated with reluctance by other physicians (86, 87). Studies including not only overweight patients may further confirm this hypothesis and explore the role of “trait” factors such as affective temperaments, personality traits, or cognitive styles on the propensity to practice regular physical activity in patients with severe mental disorders.

The following limitations of the study are hereby acknowledged. First, the inclusion of overweight patients only, which limits the generalizability of our findings to patients with different metabolic profiles. Second, the recruitment of a mixed sample of patients with severe mental disorders, which may have reduced the effect related to the diagnostic category. Third, the relatively low sample size, which does not allow us to draw firm conclusions about our findings.

In conclusion, our findings confirm that patients with severe mental disorders are sedentary and perform any type of physical activity only rarely. The recovery of patients with severe mental disorders is related to the adoption of healthy lifestyle behaviors (88–92).

Strategies aimed at increasing the levels of physical activity in patients with severe mental disorders may improve physical and mental outcomes and reduce the mortality rate. A possible way forward to improve practicing of physical activities in patients with severe mental disorders should include a specific motivational coaching on the role of exercise intervention and a personalized, patient-centered approach tailored to the needs of each individual patient.

## Data Availability Statement

Data published in this paper is available from the corresponding author upon request.

## Ethics Statement

The studies involving human participants were reviewed and approved by Ethical Committee University of Campania L. Vanvitelli. The patients/participants provided their written informed consent to participate in this study.

## Working Group Lifestyle

Vincenzo Giallonardo, Valeria Del Vecchio, Arcangelo Di Cerbo, Carlotta Brandi, Luigi Marone, Bianca Della Rocca, Department of Psychiatry, University of Campania “L. Vanvitelli”; Linda Antonella Antonucci, Giuseppe Blasi, Laura De Mastro, Francesco Massari, Giulio Pergola, Alessandra Raio, Antonio Rampino, Marianna Russo, Pierluigi Selvaggi, Angelantonio Tavella, Alessandro Bertolino, University of Bari; Alice Trabucco, University of Genoa; Ramona di Stefano, Paolo Stratta, Department of Mental Health, L'Aquila; Virginia Pedrinelli, Carlo Antonio Bertelloni, Annalisa Cordone, University of Pisa; Emanuela Bianciardi, Cinzia Niolu, University of Rome Tor Vergata.

## Author Contributions

All authors listed have made a substantial, direct, and intellectual contribution to the work and approved it for publication.

## Funding

This work was supported by the Italian Ministry of Education, Universities and Research within the framework of the Progetti di Rilevante Interesse Nazionale (PRIN) – year 2015 (Grant Number: 2015C7374S).

## Conflict of Interest

The authors declare that the research was conducted in the absence of any commercial or financial relationships that could be construed as a potential conflict of interest.

## Publisher's Note

All claims expressed in this article are solely those of the authors and do not necessarily represent those of their affiliated organizations, or those of the publisher, the editors and the reviewers. Any product that may be evaluated in this article, or claim that may be made by its manufacturer, is not guaranteed or endorsed by the publisher.
